# Publisher’s Note: Continued Publication of *Journal of Xenobiotics* by MDPI

**DOI:** 10.3390/jox10010001

**Published:** 2020-07-31

**Authors:** Franck Vazquez

**Affiliations:** MDPI, St. Alban-Anlage 66, CH-4052 Basel, Switzerland; vazquez@mdpi.com

*Journal of Xenobiotics* was launched in 2011 with the mission to publish novel research articles in the fields of the occurrence and biochemical effects of xenobiotics on all living organisms and it has been published over the past nine years by PAGEPress Publications [1]. Dr. François Gagné served as the first Editor-in-Chief [2] and he is still currently active in this role.

MDPI was founded in 1996 as a non-profit project for the promotion and preservation of the diversity of chemical compounds [3]. Since the first journal, *Molecules* [4], was launched, MDPI’s team has focused on serving academic communities’ ever growing need to quickly and openly publish the results of their research. MDPI’s portfolio has considerably expanded in the last few years, and today totals 256 journals in all fields and disciplines [5], including more than 40 in the public health area.

To ensure that we serve all academic communities well, we are delighted to take over the publication of *Journal of Xenobiotics* from PAGEPress and continue to publish the journal.

We will publish two quarterly issues in 2020, and regularly publish four quarterly issues in 2021.

Enjoy publishing in *Journal of Xenobiotics*!
